# Supplementary material for: Regulation of Vicia faba L. Response and Its Effect on Megoura crassicauda Reproduction under Zinc Stress
Source: Int J Mol Sci. 2023 Jun 2;24(11):9659. doi: 10.3390/ijms24119659 (PMC10253634; doi:10.3390/ijms24119659)
Supplement: Supplementary file 1 [file ijms-24-09659-s001.zip › ijms-2404083-supplementary.pdf]

The *Megoura crassicauda* gene sequence is as follows

>TRE

TCGAGAAAAACGGCAAATCGTACACAATGGCAAGATACTACGCACCATCGAGGGGTCC
AAGACCAGAGTCTTATAGAGAGGACTATGAATCAGCAGAATTTTTGGAAAATGAAAAT
GACAAACAAGAATTGTATACTCAAATTAAGTCTGCGGCAGAGACCGGATGGGACTTTT
CAAGTAGATGGTTTATAACTGCCAATGGCTCGGATCGTGGGGTATTGGCTGATATAAAA
ACGACGTATATAATACCAGTCGATTTGAACTGTATACTGCACAAGAATGCACAGTTGTT
GAGTACGTGGTATAGCAAGATGGGAGATAAAACTAAAGCCGAGAAATATCAGGCAATC
GCCGAGAATCTCCTCAACAGTATACAAGAAGTTATGTGGAGACCAGACCTAGGGGCGT
GGTTTGACTGGGATATGTTGAATAACAAAAGTCGAGAATATTTTTACGTTTCCAATGTT
GTACCTCTGTGGACAGAAAGCTACAACATGCCGAAAAAGGCTGTAGCTAGTTCTGTGT
TGGGATATCTAAGAGATTATCATATCATCGAAGCCGATTATTCTGTGAATTTCAACGGAA
CACCTACTTCTTTGTACAATTCATCACAACAGTGGGACTTTCCAAATGCATGGCCTCCT
CTACAAGCTTT

>TPS

GCTTACGATTTCACCGCGTTCCACATTGAGGACTACTGCTTGAACTTTATTGACTGCTG
CTGTCGGCGGCTGGGGTGCCGCGTGGACCGGACCAACATGCTGGTGGAGCTGGCCGG
ACGGACCATACAGGTGAAGGCGCTTCCGATTGGCATACCGTTCGATCGTTTCGTGCAA
CTGTCGGAGAAGGCGCCAGCGTTCCTAAACCTGTCAGAAGATGTCAAGGTGATACTGG
GCGTGGACAGGCTAGACTACACCAAGGGTCTGGTGCACAGGATCCTGGCGTTTGAGA
AATTCCTGGACAAGTATCCGGAGCACCGGGAGAAAGTGGTGCTGCTGCAGATCTCGGT
GCCGTCCAGGACGGACGTCAAGGAGTACAAGCAGCTCAAGGACGAGACTGAGCTGCT
GATCGGCCGCATCAACGGTAGGTTCTCGATGCCCAACTGGTCGCCGATTCGGTACATAT
ACGGGTGCTTGAGTCAGGAGCAGCTGGCCGCCCTGTACCGGGACTGCGCAGTGGCGT
TGGTAACGCCGCTCCGGGATGGCATGAACCTGGTGGCCAAGGAATTCGTGGCGTGCCA
GATCCGGACGCCAGGTGTGCTCATCCTGTCGCCGTTCGCCGGCGCTGGTGGCACCATG
CATGAGGCGCTGCTGGTCAACCCGTACGAGCTGGACGAGATGG

>Vg

CGCCGTCGTATCGTGTTATCTGGTTCAAACCAGCTTTTGCAATCGTGACCTGTTTCCGG
AGGGCGTTCAGCTCGGCTACAGATGGAGAGCGACGACGCATGTGGGTACCGTGTTCCC
TTCGGAGCACTTGACCAACTACTCAGTGGAAGCGTATTTTTTAGTTCAAAACGATCAA
AATTTCACAAACTTTCAAATCAAAGAATACAAAACGAGCGAGAACCAACAAGAGTAC
CCGTGGATCACACTGCCGTTTCGATGCGTTTACAGAGACGGCGAAGTACAAAAGTTCG
AGAGCGAAAGCGGCGACGTGACGGGATCGTTGAACATAAAAAGAGCATTAGCCACTA
TGTTTCAGCTGAAATTGGATTCCCTGAGGCGACCGTCGTTCGCCGCACAAGAGACTGG
AGTGTATGGAAAATGCAACGTTCAGTACCTTGTGACTAAAGAAAATAATAACACAAAT
GTAAAGAAAATAATCAACTTTTCGGCTTGTGACAATAAACTTGGACAACAATGGAGCA
ATACGCCGGCGTTCACGTGCCCATCTAGTTATCAAGACGGGAGTATGAGTCATAGTGTT
CGAAATTATAATTTGGACGAGATGAATGTAATTCGGTATTTGAATATTATCGGTACAGTA
GAGTTCCAACCATTCCAAGCAT
